# Colon Perforation As Initial Presentation of Refractory and Complicated Sclerosing Mesenteritis

**DOI:** 10.7759/cureus.17142

**Published:** 2021-08-13

**Authors:** Ammar Haikal, Kundana Thimmanagari

**Affiliations:** 1 Internal Medicine/Rheumatology, Hackensack Meridian Medical Center, Hackensack, USA; 2 Internal Medicine, Saint Michael's Medical Center, Newark, USA

**Keywords:** mesenteritis, sclerosing mesenteritis, colon perforation, refractory sclerosing mesenteritis, complicated sclerosing mesenteritis

## Abstract

Sclerosing mesenteritis (SM), a benign chronic fibrosing inflammatory disease of the mesentery, is a rare disease discovered in 1924. The prevalence of the disease is less than 1%. The exact etiology of the disease is not clear. It is thought that the integrity of the gastrointestinal lumen may be altered from chronic inflammatory effects. SM may be associated with autoimmune diseases, trauma, malignancy, or surgery. The most common clinical presentation is abdominal pain. Obstructive symptoms may occur. Diagnosis is made by CT abdomen and biopsy. Treatment includes surgical and immunosuppressive medications.

## Introduction

Sclerosing mesenteritis (SM) is a rare disease entity with a prevalence of less than 1% and an incidence of about 0.6-3.4% [1]. It is a broad term used to describe various inflammatory and fibrotic diseases that affect the mesentery. It is difficult to establish an accurate incidence given challenges in diagnosis [1]. Abdominal pain is the most common symptom. Other symptoms like fatigue, weight loss, loss of appetite, fever, vomiting, and constipation have been reported. It may be found incidentally on the CT abdomen with a mass-like effect. A biopsy helps establish the diagnosis [1]. Treatment is empiric and personalized especially if the stage of the disease is identified. We present a case of a patient with symptoms of acute bowel obstruction with a subsequent perforated colon at the time of presentation as a complication of the disease.

## Case presentation

**Figure 1:**
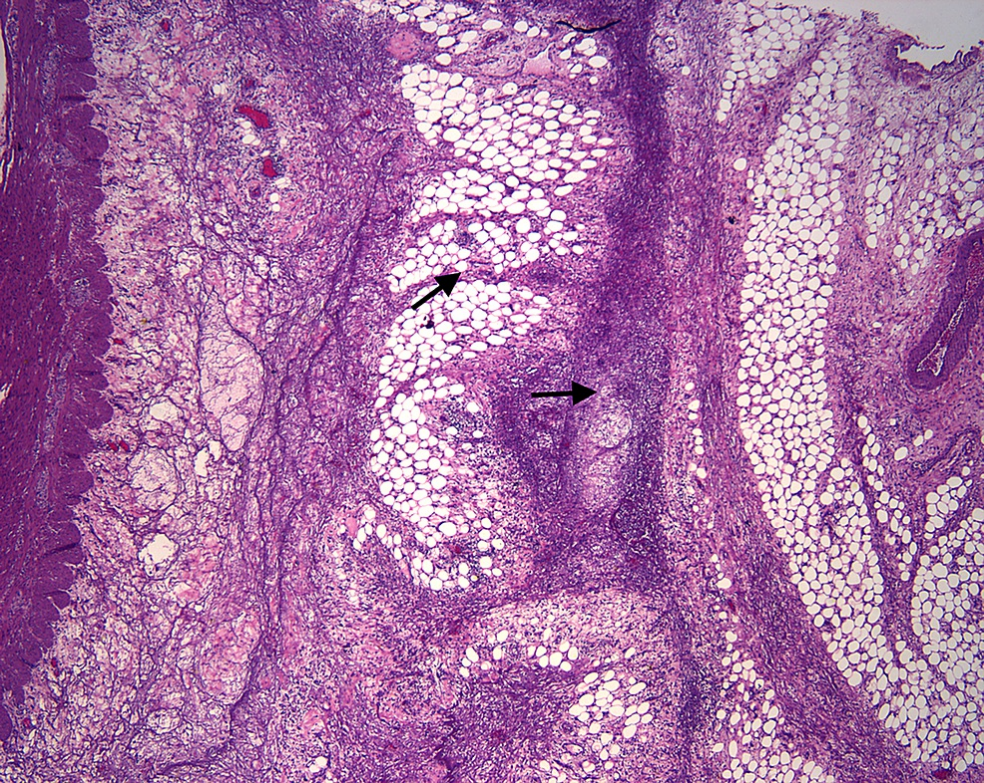
Acute and chronic inflammatory infiltrate with patchy necrosis in the mesenteric adipose tissue in a lobular pattern seen in patients with mesenteric panniculitis. There is no evidence of vasculitis , granulomas, or malignancy. The inflammatory process is only involving the mesenteric covering of the colonic tissue sparing the rest of the colonic wall including the mucosa, submucosa and the muscularis propria.

A 53-year-old Caucasian male presented in 2005 with abdominal pain and acute peritoneal signs. Initial abdomen CT showed enteritis. No mass was seen. He underwent exploratory laparotomy that revealed an inflammatory mass with a perforated colon requiring segmental resection. Pathology revealed normal mucosa of the colon, and extensive acute and chronic inflammation of the mesenteric adipose tissue with lipodystrophy (Figure 1). Foreign body or diverticulitis was not identified. He subsequently had obstructive symptoms (nausea, vomiting, and abdominal pain), and again underwent exploratory laparotomy in 2006 with right colectomy and ileo-transverse anastomosis. Pathology was like the previous sample (extensive acute and chronic inflammation in the omental adipose tissue with fat necrosis). He had several admissions for nausea, vomiting, and abdominal pain, at least once per year. CT scans performed during these episodes revealed enteritis. His colonoscopy in 2012 showed normal anastomosis, without any obstruction. Following his second biopsy and referral to rheumatology in 2014, he had received courses of prednisone which improved his symptoms. He had a recurrence of symptoms each time prednisone was decreased to below 10 mg. Azathioprine was started as a steroid-sparing agent in 2015. He remained symptom-free, and his inflammatory markers (erythrocyte sedimentation rate [ESR] and C-reactive protein [CRP]) continued to improve.

## Discussion

SM is a rare disease entity with a prevalence of less than 1% and incidence of about 0.6%-3.4% [1]. It is a broad term used to describe various inflammatory and fibrotic diseases that affect the mesentery. Pathogenesis is not clear. There are several hypotheses that may explain this inflammation. Patients who have genetic predisposition to abnormal healing and repair of connective tissue may develop SM in response to trauma, there is also associated with several autoimmune conditions like autoimmune hemolytic anemia (AIHA), systemic lupus erythematosus (SLE), Riedel thyroiditis, limited systemic sclerosis, sclerosing cholangitis, ischemia, and infection by altering vascular supply [1]. Laboratory evaluation is not specific. Elevated ESR and CRP are the most common lab findings in different reports [1]. IgG4-related SM was also reported in a child [2]. It is most commonly seen in middle age and older adults (ages 20-90 years). The rarity of this entity in childhood and adolescence has been attributed to a lesser amount of mesenteric fat in comparison to adults.

Diagnosis is made by imaging and biopsy. CT scan appears to be a sensitive method to detect radiographic features of SM. The appearance of CT findings is based on the predominant tissue finding which is fat, inflammation, or fibrosis [3]. The most common finding is either heterogeneous soft tissue mass in the mesentery in mesenteric panniculitis phase or homogenous mass in the retractile mesenteritis phase (fibrotic stage). Two radiographic characteristic appearances have been proposed to be specific findings in SM - the fat ring sign, which represents the preservation of fat nearest the mesenteric vessel, and the tumoral pseudo capsule represents a band of soft tissue separating the uninvolved mesentery from the inflamed fat, which is found in only 50% of patients [3]. However, an incidence of only 0.6% was reported in 7,620 patient studies that identified patients with SM through CT radiographic features [4]. Because of the very low detection rate on CT scans, a biopsy would be the gold standard test to diagnose SM.

Histologically, SM has three stages. The first stage is mesenteric lipodystrophy where foamy macrophages replace the mesenteric fat. Signs of acute inflammation are minimal, mostly asymptomatic with a good prognosis. The second stage, mesenteric panniculitis containing plasma cells, polymorphonuclear leukocytes, foreign body giant cells, and foamy macrophages at this point patient can present with abdominal pain, fever, malaise. The third stage is retractile mesenteritis that shows collagen deposition fibrosis, and inflammation. This collagen deposition leads to scarring, retraction of mesentery which eventually forms into abdominal masses causing obstruction of the intestine [5]. The diagnosis can be challenging as SM can mimic presentations of pancreatic cancer, fever of unknown origin, SM protein-losing enteropathy, or tuberculosis morphologically. Clinically, the most common symptoms are fever, abdominal pain, weight loss, and obstructive bowel-like symptoms. Less commonly it can also cause chylous ascites which are present in 14% of patients with SM, and bowel perforation which is a late finding [6].

Our patient had no surgical, personal, or family autoimmune history. Despite segmental resection at the time of colonic perforation, he continued to have abdominal pain and obstructive symptoms that warranted admissions over a 10-year period. He was given multiple corticosteroid courses.

Treatment is empiric and personalized especially if the stage of the disease is identified. During the first stage where fat necrosis is predominant, no treatment is necessary as it can spontaneously regress. In the second stage where chronic inflammation is present, it is indicated to treat with corticosteroids and immunosuppressants. Various immunosuppressants that have shown good results include cyclophosphamide, colchicine, azathioprine, thalidomide, and tamoxifen. In the fibrotic stage, pentoxifylline has been shown to have a good antifibrotic effect in SM [5].

Most of the published data have reported favorable outcomes with corticosteroids. According to the trial by Akram S, the use of tamoxifen and prednisone had a favorable outcome. Sixty-three percent of the patients who used tamoxifen plus prednisone had improved versus only 20% of the patients who used prednisone alone. Patients who did not respond well to prednisone alone were treated with a combination of prednisone, azathioprine, and colchicine which resulted in a similar response to tamoxifen plus prednisone. Most studies suggested the limited role of surgery unless there is a life-threatening condition such as perforation or severe obstructive symptoms [7].

Our patient had recurrent symptoms related to SM. He was referred to rheumatology for the initiation of immunosuppressive therapy. He had no other features suggestive of associated rheumatic disease. His ANA was negative. He was up to date on age-appropriate cancer screening. He was treated with prednisone course with a slow taper over three months starting at 60 mg. His symptoms reoccur every time prednisone was decreased to less than 10 mg daily. Azathioprine was added as a corticosteroid-sparing agent, and his inflammatory markers improved. He had no recurrent symptoms for about seven months.

## Conclusions

This case illustrates bowel perforation which is one of the extremely rare presentations and complications of SM. It also underscores the importance of biopsy as several CT scans that patient underwent did not show mass effect or typical radiologic features of SM. For those who present with rare complications such as perforation, a high clinical suspicion of SM is needed especially when bowel obstruction is excluded by colonoscopies. Biopsy should be performed, and if the patient is not a surgical candidate, immunosuppressive therapy should be started. Early diagnosis and treatment may decrease mortality.
